# Beyond Green: How Urban Greenspace and Extreme Climate Shape Health Outcomes in China

**DOI:** 10.1093/geroni/igaf122.246

**Published:** 2025-12-31

**Authors:** Linjiang Wei, Shujun Chai, Liangwen Zhang, Ya Fang, Xinyi Wang, Ying Han, Wen Zhang

**Affiliations:** Xiamen University, Xiamen, Fujian, China (People’s Republic); Renmin University of China, Beijing, Beijing, China (People’s Republic); Xiamen University, Xiamen, Fujian, China (People’s Republic); Xiamen University, Xiamen, Fujian, China (People’s Republic); Xiamen University, Xiamen, Fujian, China (People’s Republic); Wenzhou Medical University, Wenzhou, Zhejiang, China (People’s Republic); Xiamen University, Xiamen, Fujian, China (People’s Republic)

## Abstract

Against the dual backdrop of climate change and rapid urbanization, a thorough analysis of the spatiotemporal interactions between extreme climate events, urban green spaces, and health outcomes in aging populations holds significant theoretical and practical importance. This study innovatively employs the multiscale geographically and temporally weighted regression (MGTWR) model to systematically explore the mechanisms through which extreme climate exposure and greenness coverage influence the prevalence of chronic diseases and comorbidities among urban older adults (age ≥60) in China. By integrating data from two national cohort studies (CLASS and CHARLS, 2015-2020), along with multidimensional indicators such as meteorological data, urban greenness metrics, the study yields the following key findings: First, extreme climate exposure exhibits significant spatial autocorrelation, with its impact on chronic disease comorbidity prevalence showing a spatial gradient that increases from southwest to northeast (standardized β > 0.24). Second, urban greenness demonstrates dual health effects: park greenness significantly reduces chronic disease prevalence through its ecological regulation functions, but may increase short-term comorbidity risks due to factors such as behavioral changes (τ = 0.2). Third, the MGTWR model (R² = 0.772) outperforms traditional methods in terms of goodness-of-fit, effectively revealing the spatiotemporal heterogeneity of health-environment relationships. Based on these findings, the study proposes policy recommendations, including establishing a climate-resilient health protection system, optimizing the precise allocation of green space health services, and improving the framework for environmental health governance. This research offers critical guidance for addressing the dual challenges of chronic disease burden and climate vulnerability in the context of rapid urbanization.

